# NovoSorb® Biodegradable Temporizing Matrix: a novel approach for treatment of extremity avulsion injuries in children

**DOI:** 10.1007/s00068-024-02535-6

**Published:** 2024-04-26

**Authors:** Safiullah Najem, Miriam Fattouh, Kristofer Wintges, Benjamin Schoof, Merle Koerner, Konrad Reinshagen, Ingo Koenigs

**Affiliations:** 1 Department of Pediatric Surgery, University Medical Center Hamburg-Eppendorf (UKE), Martinistrasse 52, 20251 Hamburg, Germany; 2 Department of Pediatric Surgery, Altona Children’s Hospital, Bleickenallee 38, 22763 Hamburg, Germany; 3 Department of Trauma Surgery and Orthopedics, University Medical Center Hamburg-Eppendorf (UKE), Martinistrasse 52, 20251 Hamburg, Germany

**Keywords:** BTM, Dermal skin substitutes, Templates, Pediatric, Avulsion injury, Wound therapy

## Abstract

**Purpose:**

In pediatric population, large soft tissue defects occur in avulsion injuries. In addition to the challenges of primary surgical therapy, elasticity, appearance and function of the scar in children are of crucial importance, especially in the context of body growth. So far various flaps, plasties, skin grafts and dermal substitutes have become established, although infections and skin shrinkage remain challenging. In 2020, a new skin substitute material—NovoSorb® Biodegradable Temporizing Matrix (BTM)—was introduced in Europe for temporary wound closure and tissue regeneration. The aim of this study was to evaluate the value of BTM in pediatric patients.

**Methods:**

The study included all children treated with BTM after traumatic soft tissue defects following limb avulsion injuries between June 2021 and June 2023 at a university hospital.

**Results:**

7 patients with limb avulsion injuries were treated with BTM, 4 boys, 3 girls. Mean age was 6.5 years (2–11 years) at the time of BTM placement. 4/7 had concomitant fractures. BTM was used successfully in all cases, infection did not occur, skin shrinkage was seen in one case. Split thickness skin graft (STSG) after BTM application was performed in average after 33 days (26 to 39 days). Limitations of this study were highlighted.

**Conclusion:**

BTM is a promising alternative for reconstruction of complex trauma extremity wounds in children following avulsion injuries, even in cases of concomitant bone injuries. Interpretation may be limited by sample size.

## Background

Decollements of extremities are rare in pediatric population, but often result in deep soft tissue defects requiring specialized plastic surgical treatment to obtain optimal functional and aesthetic results [1]. Established reconstructive surgical options for covering soft tissue defects in children include replantation, flaps, split-thickness skin grafts (STSG), tissue expansion and use of dermal skin substitutes (DSS) [2, 3]. Petrie et al. summarized eleven commercially available acellular skin matrices of human, bovine or porcine origin in a review [4]. In addition to STSG, flaps or permanent artificial skin substitutes, "Self-Assembled Skin Substitutes (SASS)", have also been described for extensive skin defects or in burns without sufficient donor areas [5, 6]. In cases of concomitant injuries such as fractures or tendon injuries, the type of osteosynthesis or immobilization can influence wound treatment selection. In most strategies, the affected avital cutis or subcutis is removed. In order to reduce the final wound size, as much tissue as possible should be preserved. Autologous STSG is used for covering partial dermal or dermal defects. It is advantageous for rapid closure and low risk of infection, but disadvantageous for high rate of hypertrophic scar formation and contraction [7–9]. Over the last 50 years, tissue expansion has developed into a reliable reconstructive method, which is, however, associated with a high complication rate [10, 11]. Flaps are used for defect covering surgery with favorable results and mostly excellent functional outcome despite the balancing act between flap thickness and flap thinning. It remains associated residual risks of flap necrosis, non-union, wound infection, dehiscence and moderate aesthetic outcome [7, 12–14]. In this context, biologically or synthetically engineered dermal skin substitutes were introduced as a novel option in the 1980s. The dermal substitute Integra® has been used primarily in children since the early 2000s and is now an established dermis replacement procedure in children [1, 2, 15–20]. A limitation of Integra® is the occurrence of skin shrinkage and infection. Greenwood et al. introduced a novel dermal template NovoSorb® Biodegradable Temporizing Matrix (BTM) in 2012 [19, 21–25]. In 2016, Ceshire et al. published a comparative study on BTM and Integra® for temporary wound closure in mice [17]. Subsequently, several studies showed the successful use of BTM in adults, mainly in treatment of burns [18, 26]. In children, only few case reports describe the use of BTM in burns [5]. To date, only three pediatric cases have been published with BTM in reconstructive surgery [25, 27, 28].

BTM is a biodegradable polyurethane foam with a temporary non-biodegradable polyurethane sealing membrane as a wound barrier and secretion drainage function (https://polynovo.com/product-btm). After application of BTM to the wound bed, cell migration, proliferation and capillary filling take place through the BTM porous matrix. This is followed by the degradation of the BTM matrix [26]. Converting a suitable defect wound bed for STSG is one of BTMs major advantages [18]. Subsequently, STSG transplantation is performed on the “neo-dermis” [5]. Lo et al. confirmed that long-term scar quality in adult burn patients is very satisfactory [29]. Wu-Fienberg studied six adult patients with upper extremity trauma treated with BTM over a period of one year and presented convincing results [30].

In pediatric reconstructive plastic surgery, BTM is not widely known. To date, clinical data for children is limited: only two single pediatric case reports of decollement reconstructions are published. The first on a 7-year-old child with a lower limb degloving injury who was run over by a truck [27], and the second on a 15-year-old boy with a multiplanar upper limb degloving injury [28]. In particular, there is still insufficient data on the incidence of infection, skin shrinkage and functional outcome after two-stage BTM-STSG-treatment in children. Therefore, the research aim was to analyze retrospectively the pediatric patients treated with BTM for extremity traumatic soft tissue defects.

## Methods

The study is a longitudinal observational study designed to evaluate a new therapeutic approach for reconstruction following tissue loss due to avulsion trauma of extremities. The aim is to investigate the viability of the new technique by analyzing the reconstructive outcome and demonstrating reproducibility and clinical benefit in children. Inclusion criteria were all pediatric patients (≤ 18 years of age, hereafter referred to as children) undergoing BTM and STSG treatment of skin soft tissue defects of extremities following avulsion injuries in a period between June 2021 and June 2023. Patients were excluded from the study if they did not have an avulsion injury with significant loss of tissue with required necessity of defect coverage or alternative treatment. BTM application and STSG transplantations were performed by the same two pediatric surgeons and traumatologists. Patient demographics and surgical details were included in the analysis. Exemplary two cases are presented in detail.

All procedures were performed under general anesthesia. Prior to BTM application, all pediatric patients underwent radical surgical wound debridement with avital tissue removal after demarcation, and the wound was temporarily conditioned with vacuum dressing with a continuous negative pressure of 125 mmHg (negative pressure wound therapy (NPWT)). A KCI V.A.C system with a Granufoam silver dressing kit was used (KCI Medizinprodukte GmbH, Wiesbaden, Germany). Once the wound had achieved a vital wound bed and the wound margins were clear, BTM was applied. NovoSorb® BTM is commercially available (Polymedics Innovations GmbH, Denkendorf, Germany). BTM was fixed at the wound margins. The applied BTM was optionally covered with a vacuum dressing or fatty gauze. In case of NPWT, vacuum dressing was changed every 5 to 10 days. When macroscopic vascularization of the BTM matrix was visible, delamination of the sealing membrane and definitive defect coverage were performed using autologous 0.1 mm thin STSG, generally non-meshed. The favored donor-site was the hairy scalp unless otherwise preferred by patient or parents. A STSG take rate more than 95% was defined as healing.

Wound swabs were taken to assess the microbiological colonization of the wound at time of BTM application and prior to STSG. A perioperative antibiotic prophylaxis was administered regularly, patient received antimicrobial therapy if a pathogen was detected. Photographic wound documentation was performed. Patients were seen as outpatients at least every three months for follow-up.

## Results

A total of seven patients with traumatic avulsion injuries were treated with BTM during the two-year study period. The included wounds resulted of avulsion trauma and were localized at the extremities, the majority were localized on the upper extremity (5/7). The traumas were caused by trucks (4/7), by SUV (2/7) and sports device (1/7). Of the included patients, three were female and four were male. At the time of BTM placement, the average age was 6.5 (2 to 11 years). All patients had no secondary diseases. All wounds showed full-thickness tissue defects without possibility of primary closure. The average defect size was 180 cm^2^. At time of BTM-placement, a distinct pathogen was isolated in three wounds. Average time from BTM application to definitive defect coverage by STSG was 33 days, with a range from 26 to 39 days. BTM was successfully integrated in all cases and no infections were observed. Total duration of NPWT was 13.3 days (2 to 43 days) before BTM application and 5.3 days (0 to 18 days) after BTM application. Details are shown in Table 1. In the following, patient number 1 and 2 are presented in detail. Further information for patient number 3–7 is displayed in Table 1 and Fig. 3.

**Table 1:** Overview of seven patients with traumatic avulsion injury in the context of NPWT, BTM-, STSG-treatment from June 2021 to June 2023

Patient number	Patients´ age on DA *(years)*	Defect localization	Concomitant injuries	1st surgery at study department after DA *(days)*	NPWT start after DA *(days)*	Wound size BTM-A *(cm*^*2*^*)*	Pathogens detected by microbiological swabs	Donor site, meshed	STSG after BTM-A *(days)*	NPWT *[before* + *after BTM-A] (days)*	STSG take *(%)*	Total treatment DA—STSG *(days)*	Shrinkage	Infection	Follow-up *(months)*
1	10	lower limb, foot	tibia fracture	23	23	448	Stenotrophomonas maltophilia	thigh, non-meshed	34	49 [43 + 6]	100	77	none	none	28
2	6	arm	olecranon fracture, pelvic fracture	0	0	480	none	thigh, non-meshed	39	36 [18 + 18]	100	57	none	none	26
3	2	arm	none	6	6	42	none	hairy scalp, non-meshed	38	10 [6 + 4]	100	50	yes	none	25
4	11	inner ankle, foot	none	18	23	50	Staphylococcus aureus	thigh, meshed	26	7 [2 + 5]	100	51	none	none	15
5	8	upper arm	none	1	5	140	none	thigh, non-meshed	30	9 [5 + 4]	100	40	none	none	14
6	7	lower arm	olecranon fracture	0	3	90	none	hairy scalp, non-meshed	32	12 [12 + 0]	100	47	none	none	9
7	2	hand	phalanx fractures, phalanx tendon	0	0	10	Staphylococcus epidermidis	hairy scalp, non-meshed	34	7 [7 + 0]	100	41	none	none	6
Ø	6.5			6.8	8.6	180			33.3	18.6 [13.3 + 5.3]	100	51.9			17.6

*Ø* average, *BTM-A* at time of BTM application, *DA* day of accident, *NPWT* negative pressure wound therapy with a continuous suction of 125 mmHg, *STSG* split-thickness skin grafting

### Patient number 1: treatment of decollement of the right lower limb and the right foot

**Fig. 1:**
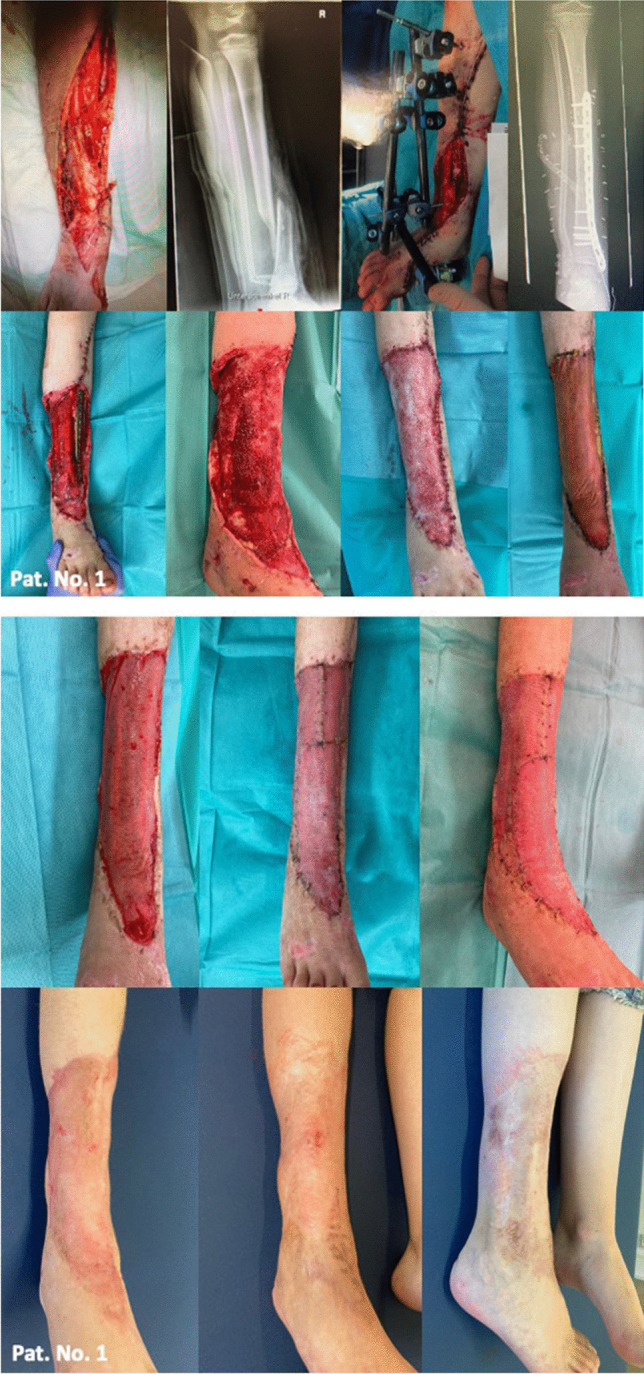
Patient number 1. A 10-year-old female with traumatic defect affecting semicircular the anterior aspect of the right lower leg and the right foot: 1. image series: Wound situation on admission at primary hospital, tibia fracture, external fixator and plate osteosynthesis of the tibia 2. image series: Before and after removal of the plate, BTM application. 3. image series: Successful integration of BTM, definitive defect coverage with STSG. 4. image series: Satisfactory scar pattern and skin elasticity in a 3- (left) and 22-month (right) follow-up

A ten-year-old female was referred from a tertiary hospital for further surgical treatment after sustaining an avulsion injury by a reversing garbage truck. The right lower leg was affected semi-circularly ventral and the right foot semi-circularly dorsal by the accident. The patient had a type 3B according to the Gustilo-Anderson classification open fracture of the right tibia, which was primarily treated with open reduction and external fixator and finally, eight days later with a plate osteosynthesis of the right tibia (Fig. 1). The decollement was initially treated with NPWT. Eight days later a latissimus dorsi flap and an autologous STSG were performed, unfortunately an infection occurred and flap and STSG were lost. At this point the patient had been transferred to our department. After several surgical debridements on day 43 of NPWT, the plate could be removed, the remaining skin soft tissue defect measured 448 cm^2^ and was covered with BTM.

*Stenotrophomonas maltophilia* colonization was detected at the time of BTM application. Therefore, the patient received antimicrobial therapy. NPWT was finished after 6 days. 34 days after BTM application the child was readmitted. Definitive defect coverage with non-meshed STSG could be performed with the left thigh as the donor site, similar to the previous surgery at the primary hospital. BTM and STSG integrated completely. In a more than two-year-follow-up the patient was completely mobile, showed a satisfactory scar pattern and skin elasticity. The patient was able to resume her favorite sports like gymnastics and basketball.

### Patient number 2: treatment of decollement of the left arm

**Fig. 2:**
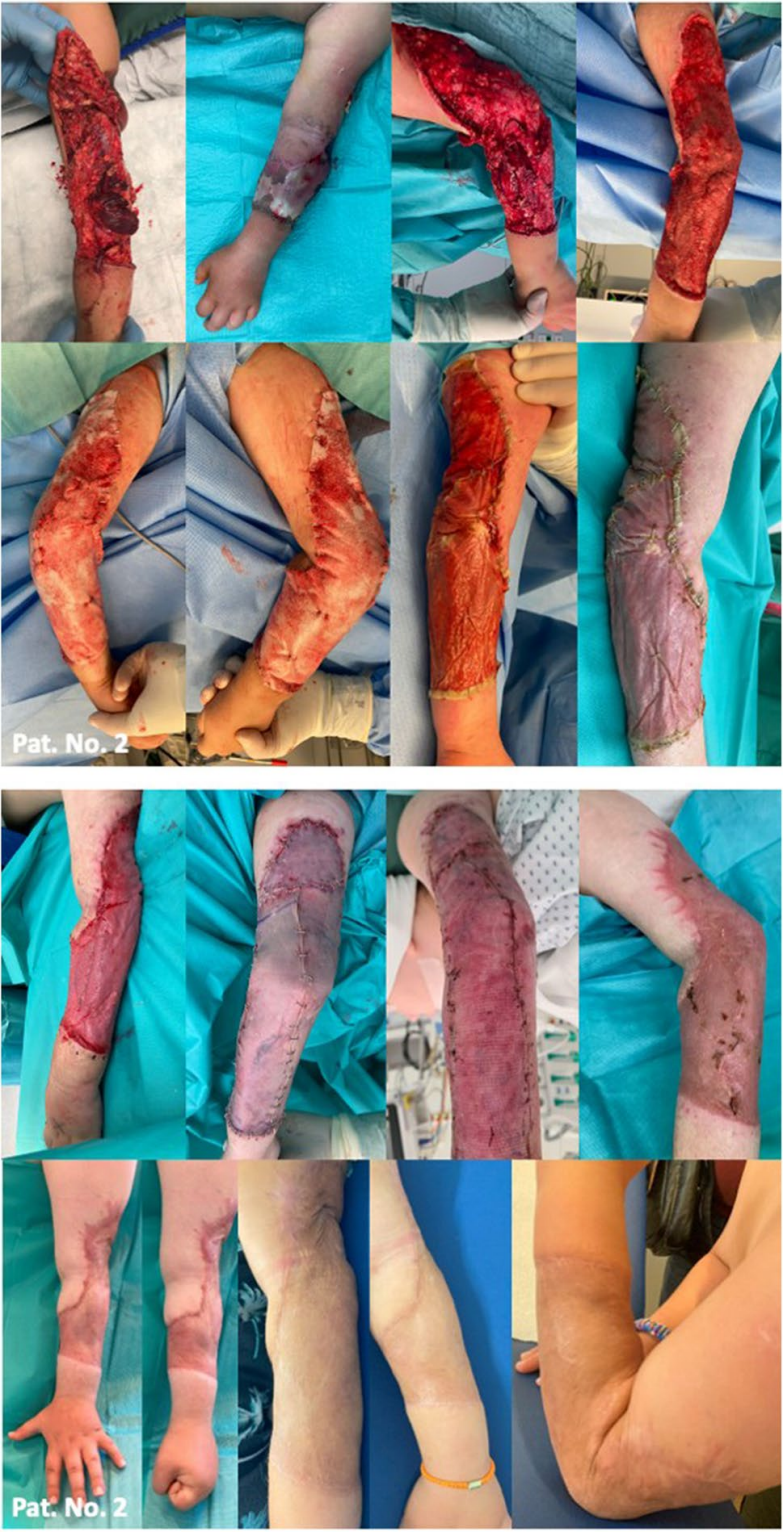
Patient number 2: A 6-year-old male with traumatic defect affecting semicircular the anterior lateral aspect of the left arm: 1. image series: Wound situation on admission and after final debridement, 2. image series: BTM application and remodelling. 3. image series: Successful integration of BTM, definitive defect coverage with STSG. 4. image series: Satisfactory scar pattern and skin elasticity in a 6- (left), 12- (middle) and 21-month (right) follow-up

A six-year-old male was admitted to our emergency department due to an avulsion injury caused by a garbage truck. The skin tissue defect affected the left arm (Fig. 2). In course of the accident, the patient sustained a type 3B according to the Gustilo-Anderson classification open non-displaced olecranon fracture on the left side and a pelvic fracture with concomitant dissection-related stenosis of the left femoral artery requiring venous patch-plasty as well as a Morel-Lavallée lesion of the coccyx region. Emergency closed reduction of the anterior and posterior pelvic rings and osteosynthesis with a supraacetabular external fixator was performed. The olecranon fracture was treated conservatively, the sacral defect following the Morel-Lavallée lesion with NPWT and subsequent flap surgery. At time of BTM application, the arm´s defect size measured 480 cm^2^. A microbiological pathogen was not found. NPWT was finished after 18 days of BTM. 39 days after BTM application non-meshed STSG from the left thigh (according to patient´s and parent´s request) could be achieved. The STSG take was 100%. In a 26-months-follow-up, the patient showed a satisfactory scar pattern and skin elasticity and was able to actively participate in physical education classes and pursue his favorite sports again (Fig. 2).

**Fig. 3:**
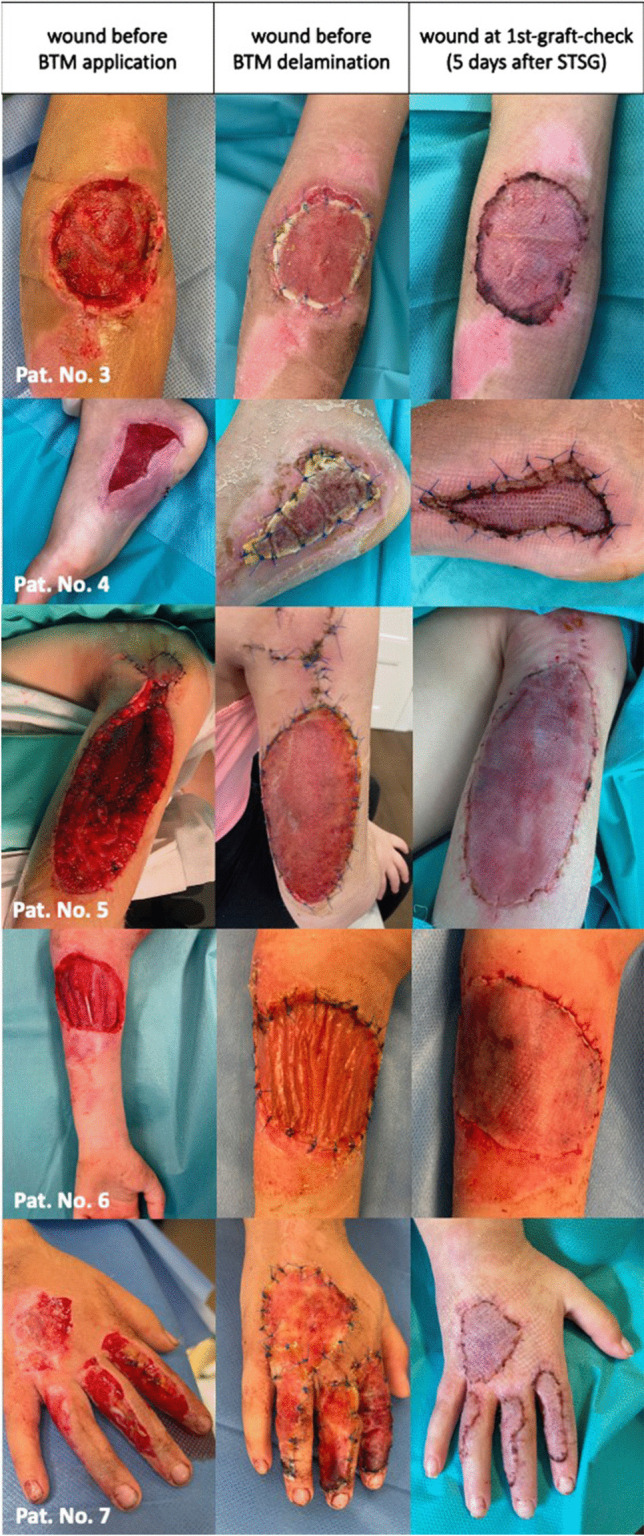
Brief summary of patient number 3 to 7 with avulsion injury Pat. No. 3 A 2-year-old male with defect affecting the lateral aspect of the left arm. Pat. No. 4 A 11-year-old male with defect affecting the inner ankle of the left foot. Pat. No. 5 An 8-year-old female with defect affecting semicircular the posterior aspect of the right upper arm. Pat. No. 6 A 7-year-old male with defect of the flexion-side of the left lower arm semi-circularly. A type 3A according to the Gustilo-Anderson classification open displaced olecranon fracture was treated with Kirschner-wire-fixation after initial external fixation. Pat. No. 7 A 2-year-old female with defect of dorsal part of the right hand with additional extensor tendon injuries (Verdan zone 4) as well as a type 3A according to the Gustilo-Anderson classification non-displaced proximal phalanx fracture of the right index and middle finger. The tendon was reconstructed, the fractures treated conservatively

**Fig. 4:**
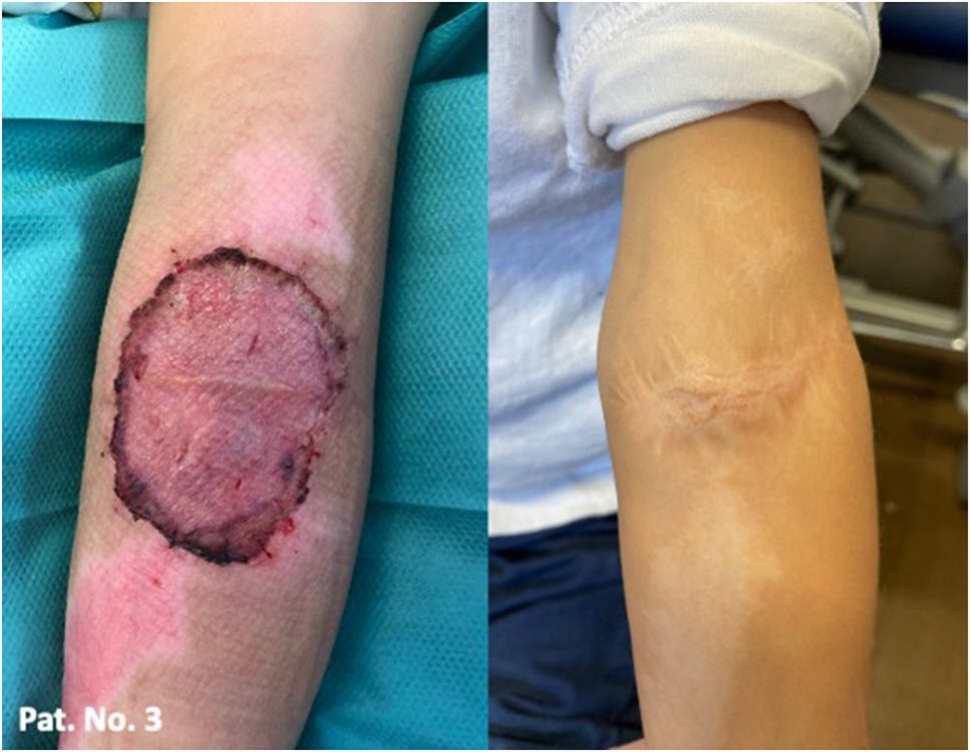
Patient number 3: A 2-year-old male with an avulsion injury of the left arm: Left image: Successful STSG take. Right image: Postoperative graft skin shrinkage in a 16-month follow-up

Cases 3–7 are summarized in Fig. 3. Only in patient number 3 skin shrinkage was determined after a 16 months follow-up (Fig. 4). However, no functional limitations occurred. Nine months later, there were no movement restrictions, on the contrary, favorable aesthetic outcome with a significantly smaller scar due to shrinkage could be observed.

All patients had a satisfactory scar pattern and skin elasticity without any functional impairment at the end of the follow-up period.

## Discussion

Since the first description of the use of BTM in humans by Greenwood and Wagstaff in 2012, treatment with BTM has been reported for various indications: Burns, open soft tissue infections, traumatic and dermatological lesions, chronic wounds and ulcers [21, 31, 32]. The majority of cases are published for burns, but BTM is becoming increasingly important in reconstructive surgery in adults [26, 33]. In children, the indications for BTM in reconstructive soft tissue surgery need to be reviewed, as only three cases have been published to date [25, 27, 28].

In the present study, BTM application proved to be a reliable and versatile reconstructive procedure for pediatric patients with extremity avulsion injuries. Overall, all patients were successfully treated with BTM and grafted with autologous STSG 40 to 77 days after the injury and 30 to 39 days after BTM application. The proposed donor site was the hairy scalp as the “hidden donor site” [34]. Other donor sites were chosen mainly by older children for different reasons, e.g. patient number 1 (at previous donor site after grafting at the primary hospital). In all cases, the treatment was successful with functionally and aesthetically sufficient closure after an appropriate treatment time. These results go hand in hand with the published literature describing BTM as a fully synthetic option for the treatment of deep tissue defects in adults and burns [29]. All patients in the study underwent the two-stage surgical procedure described in detail for adults [26, 31, 32, 35].

Secondary wound healing after BTM treatment without the need of STSG was reported by Li et al. and Granick et al. [18, 31]. For relevant defect sizes, the value of secondary wound healing regarding healing time and scar quality should be carefully scrutinized. In all previously published reconstructive pediatric cases, a STSG similar to our protocol was performed [25, 27, 28]. Similar to these reports, STSG should be performed after BTM to achieve sufficient and high quality final wound closure.

Compared to other dermal substitutes, such as Integra® or cadaveric allografts, BTM has been shown to have a low complication profile in wound models in mice and pigs or in adult burns: significantly lower infection rates, lower rates of STSG shrinkage, contracture formation and high long term scar quality [17, 21, 29, 35, 36]. Clinically relevant tissue infections described after BTM application could not be reproduced in the present study [33]. Even in cases of BTM infection good results without distinct loss of relevant BTM areas are reported regularly [33]. This may be a relevant difference to Integra®, where contrary to this a loss of huge areas of Integra® is seen clinically often in occurrence of infections [25]. In mice model both templates were inaugurated similarly, but in BTM a higher neovascularization and inflammatory response was demonstrated, which may prevent infections [17].

More than half of the study patients in our cohort had a sterile wound swab taken prior to BTM application. A total of three different pathogen strains were detected, however, no infection occurred in these patients. Antibiotic treatment is not necessary from a clinical point of view, but due to our limited experience during the study period, we decided to administer antibiotic therapy in the event of pathogen detection. Noteworthy we did not observe any clinically relevant infections. Whereas in one case, we observed postoperative shrinkage (Fig. 4). This occurred in a smaller affected area without extending over a joint, resulting clinically in an even smaller scar without any functional limitation. In all larger defects with joint involvement, we observed no shrinkage and no impairment of movement with good elasticity. Unfortunately, no cutometry was performed due to absence of the device. Shrinkage is the most common complication of large wound closure after infection [7]. It leads to unfavorable aesthetic results and can lead to functional impairment, especially across joint. Reid et al. published in 2007 that wounds treated with STSG in a porcine model shrank more than those treated with artificial skin substitutes [37]. In an analysis between Integra® and BTM in a mouse model, Chesire et al. described similar wound closure using BTM or Integra® without signs of wound contracture [17].

BTM has been shown to be a reliable and simple reconstructive option to avoid complex reconstructive surgery, especially in younger growing infants with large defects and/or open tendon/bone injuries [18, 30, 38]. The major benefit of BTM compared to complex flap surgery is the simple technical feasibility due to the greater challenges in surgical flap technique at a young age, as the vessel diameter is a limiting factor in children [39]. The average age in our study group was 6.5 years, so we therefore suggest that BTM is also suitable for toddlers and infants. Similarly, in another context, we were able to successfully perform a BTM application in a preterm at the age of 28 weeks of gestation with a body weight of 700 g. Age therefore does not appear to be a limit for the use of BTM.

It is still an important question how long and whether NPWT is necessary. In everyday clinical practice, NPWT often appears to be used as a safety system for patients, relatives and physicians. On the other hand, several studies confirmed that the combination of Integra® and NPWT, artificial dermal skin substitute and NPWT or BTM and NPWT led to improve rates of its integration and skin graft take [40–42]. Other described advantages of BTM included early mobilization through physiotherapy and regaining partial sensation over the majority of the wound [43]. In the present study, the sensation of the reconstructed area was clinically quantified and satisfactory. For this specific question further diagnostic studies in children are required.

In our cohort, the overall treatment duration averaged 51.9 days, although the individual duration appeared to decrease during the study period. This is certainly related to the type and size of the injury, as well as the localization and the exposed structures, but also reflects to some extent our learning curve and ongoing experience with BTM. Others reported a shorter overall-treatment-time in adults with a minimum of 27 days [28, 30]. It is possible that the total treatment time may be reduced somewhat in the future, but due to the time the BTM requires to convert adequately, the total time will certainly not be less than 30–40 days including STSG.

## Limitations of the study

The following limitations should be taken into account when evaluating the study:

1. The rarity of the disease and the correspondingly small study population.
2. All patients were recruited retrospectively at the pediatric surgery department of the study authors. A prospective, randomized, multicenter national or global study would be very demanding but desirable to proof this promising results.
3. No direct comparisons with other wound closure methods were made in this study.

## Conclusions

To our knowledge, this is the first study of NovoSorb® Biodegradable Temporizing Matrix in complex extremity wounds in a pediatric population. Our results show that BTM application appears to be a promising alternative option in pediatric reconstructive surgery to avoid multiple complex surgery in the growing body, particularly for larger soft tissue defects: low infection and low shrinkage rate, usability even in case of open tendon and bone injuries, early mobilization by means of physiotherapy and absence of religious or cultural limitations due to its synthetic composition. We are aware of the small study population, but we consider that our results – more empirical than scientific data – have a scope for the establishment of BTM in extremity decollement surgery in children. Therefore, further prospective comparative research in larger cohorts is needed.

## Data Availability

The data that support the findings of this study are available on request from the corresponding author. Data are not publicly available due to the privacy of participants.

## Abbreviations

BTM: Biodegradable temporizing matrix
DSS: Dermal skin substitutes
NPWT: Negative pressure wound therapy
SASS: Self-assembled skin substitute
STSG: Split thickness skin graft
